# The neoteny goldilocks zone: The evolution of neoteny in *Ambystoma*

**DOI:** 10.1002/ece3.11240

**Published:** 2024-04-08

**Authors:** Thom A. Lyons, Kevin Arbuckle

**Affiliations:** ^1^ Department of Biosciences, Faculty of Science and Engineering Swansea University Swansea UK

**Keywords:** amphibian ecology, axolotl, life history evolution, Paedomorphosis, phylogenetic comparative methods

## Abstract

Neoteny is a developmental strategy wherein an organism reaches sexual maturity without associated adult characteristics. In salamanders, neoteny takes the form of individuals retaining aquatic larval characteristics such as external gills upon maturation. Mole salamanders (*Ambystoma*) occupy a wide range of habitats and areas across the North American continent, and display examples of non‐neotenic, facultatively neotenic and obligate neotenic species, providing high variation for investigating the factors influencing the evolution of neoteny. Here, we use phylogenetic comparative methods to test existing hypotheses that neoteny is associated with elevational and latitudinal distribution, cave‐associated isolation, and hybridisation‐related polyploidy. We also test if neoteny influences the diversity of habitats a species can occupy, since the restriction to an aquatic life should constrain the availability of different niches. We find that neoteny tends to occur in a narrow latitudinal band between 20–30° North, with particularly narrow latitudinal ranges for obligate compared to facultative neotenic species (16–52° North). We also find that facultatively neotenic species occur at elevations more than twice as high as other species on average, and that species with a higher frequency of neoteny typically have lower habitat diversity. Our results suggest that evolutionary transitions between non‐neotenic and facultative neoteny states occur at relatively high and approximately equal rates. Moreover, we estimate that obligate neoteny cannot evolve directly from non‐neotenic species (and vice versa), such that facultative neoteny acts as an evolutionary ‘stepping stone’ to and from obligate neoteny. However, our transition rate estimates suggest that obligate neoteny is lost >4‐times faster than it evolves, partly explaining the rarity of obligate species. These results support the hypothesis that low latitudes favour the evolution of neoteny, presumably linked to more stable (aquatic) environments due to reduced seasonality, but once evolved it may constrain the diversity of habitats.

## Graphical abstract

Neoteny is a condition by which an organism reaches sexual maturity while retaining juvenile characteristics. Here, we use comparative phylogenetic methods to test existing theories into the drivers of neoteny in Ambystoma and whether neoteny may influence the diversity of habitats a species can inhabit. Our results suggest neoteny tends to occur in a narrow latitudinal band between 20–30° North, and that facultatively neotenic species occur at elevations more than twice as high as other species on average, and that species with a higher frequency of neoteny typically have lower habitat diversity.

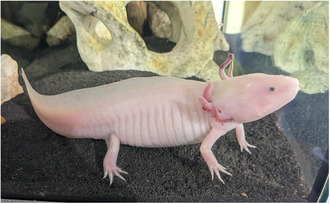

## 1 INTRODUCTION

**FIGURE 1:**
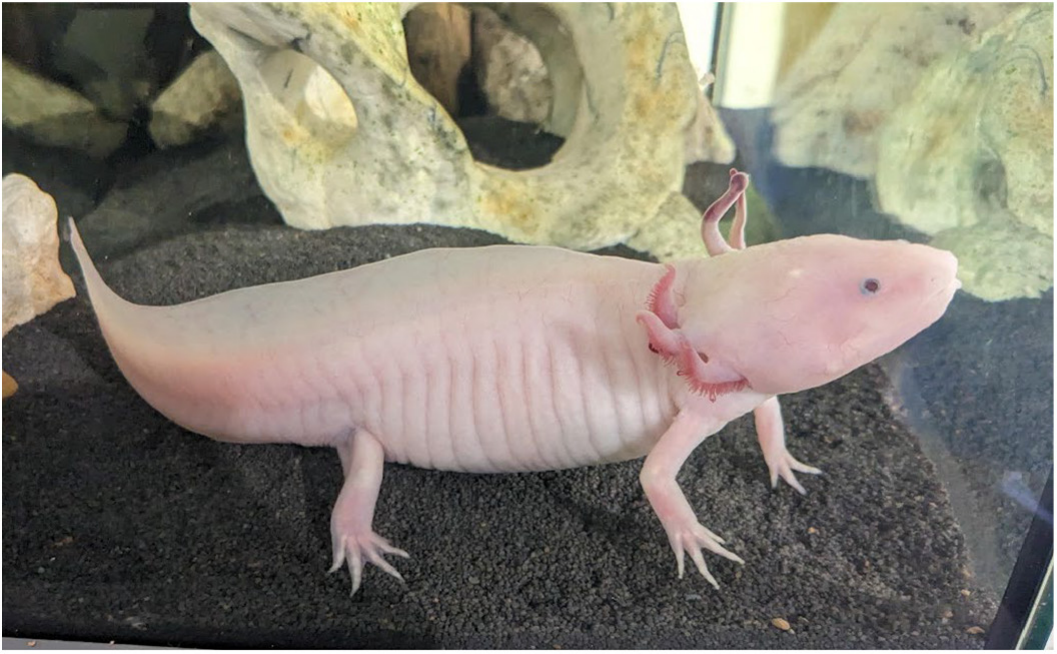
*Ambystoma mexicanum*, an obligate neotenic species showing the external gills and flattened paddle tail characteristic of a neotenic salamander. *Note*: this is a captive albino specimen (wild specimens are dark brown dorsolaterally and off‐white ventrally) but other than coloration the morphology is species‐typical.

Neoteny is a developmental strategy characterised by the retention of juvenile morphology after sexual maturation (Bufill et al., 2011; Gould, 1985). Neoteny is especially prevalent and well documented in salamanders; indeed, Kollmann (1885) first coined the phrase neoteny while studying a group of Mexican salamanders (*Ambystoma mexicanus*) which retain juvenile features such as larval gills, paddle tail and aquatic lifestyle into adulthood (Figure 1). In salamanders, retention of larval features after sexual maturity is linked to a lack of thyroxine driven metamorphosis (Rosenkilde & Ussing, 2004), but the environmental and ecological factors associated with the evolution of neoteny remain largely unknown.

*Ambystoma* (mole salamanders) is an excellent model genus to study the evolution of neoteny. The species within this genus are widespread across a large variety of habitats ranging from Canadian boreal forests to the dry mountains of central Mexico (AmphibiaWeb.org, 2023; IUCN Red List 2022). Mole salamanders show substantial diversity of neotenic states across the 32 species, with many species always undergoing metamorphosis (non‐neotenic), some rarely or never metamorphosing (obligate neotenic), and some having variation of metamorphosing and neotenic individuals or populations (facultatively neotenic) (Everson et al., 2021). In facultatively neotenic populations, mating often occurs between neotenic and non‐neotenic individuals, confirming that these two alternative developmental strategies may occur within a population of a given species and do not intrinsically represent genetic isolation from terrestrial counterparts (Everson et al., 2021).

Several hypotheses have been proposed to explain the ecological drivers of the evolution of neotenic salamanders (Bonett et al., 2022), though these remain to be tested in an interspecific framework. Here, we introduce four key ideas from previous literature which we investigate in a phylogenetic comparative analysis of *Ambystoma*.

Neotenic individuals have been reported to be more prevalent at higher elevation in populations of the facultatively neotenic species *A. gracile* (Snyder, 1956) and *A. tigrinum* (McKean et al., 2002). This may be related to cooler temperatures at higher elevations, since low temperatures are known to inhibit thyroxine‐driven metamorphosis in other salamanders (Moriya, 1983). Higher altitudes are also characterised by higher precipitation because air cools as it rises, so higher elevation distributions may have more permanent bodies of water and oxygen rich flowing streams. Such an increase in wetland habitats has been proposed to be selectively advantageous to neotenic salamanders, which are restricted to water due to the presence of gills (Schoch & Fröbisch, 2006; Snyder, 1956). Mountains can also pose challenges to overground movement, with unfavourable terrestrial conditions (e.g. lower temperatures and resource availability) and this may cause isolation of neotenic populations from terrestrial counterparts, possibly resulting in speciation (Everson et al., 2021).

Similar to altitudinal patterns, temperature also decreases with higher latitudes and so neotenic species may be expected to be more common further from the equator. However, lower latitudes also experience more stable temperatures throughout the year due to reduced seasonality (Teeri & Stowe, 1976). Since amphibian metamorphosis is generally triggered by seasonal changes in temperature, daylength and rainfall (Bonett et al., 2022; Semlitsch & Wilbur, 1988; Werner, 1986), and more stable conditions might convey reduced risk of water bodies drying up in summer months, a contrasting prediction that neoteny will be more likely to occur at lower latitudes has also been suggested (Bonett et al., 2022).

Within *Ambystoma* there exists a hybrid complex of polyploid all female populations, the *Ambystoma jeffersonianum* complex. Salamanders from this group of species will ‘steal’ sperm packages from other *Ambystoma* species and produce klepton offspring that will gain an additional chromosome set from the sexually parasitised male species (Macgregor & Uzzell, 1964; Uzzell, 1964). This has resulted in polyploid individuals from this species complex with up to five complete chromosome sets (Bogart & Lichts, 1986). Many salamanders have unusually large genomes, and this attribute is thought to be linked with the tendency of a species to metamorphose, since some neotenic species have notably larger genomes than metamorphosing species (Bonett et al., 2022). It may be that the large genome sizes of these polyploid hybrids are linked with inhibited metamorphosis, although the mechanism for the proposed relationship remains unclear. We therefore test the hypothesis that this kleptogenesis‐induced polyploidy may be associated with the evolution of neoteny in *Ambystoma*.

Neotenic salamanders from other families, such as the olm (*Proteus anguinus*), are commonly found in subterranean habitats such as caves (Culver & Pipan, 2019). Caves may provide a build up for neotenic genes via strong selection against terrestrial individuals that are swept into the cave, which may be unable to escape and hence drown. Therefore, in cave environments, accumulation and persistence of individuals genetically disposed to neoteny may cause these genes to become fixed over time. Furthermore, caves would isolate this population from the wider gene pool and cause neoteny to be increasingly fixed over time. For this reason, it has been proposed that neotenic individuals may be disproportionately found in these subterrain aquatic ecosystems (Barr & Holsinger, 1985; Jones & Thompson, 1983).

Once neoteny evolves, it could have implications for the diversity of habitats available to the species. We might expect that populations of salamanders that possess both terrestrial and aquatic adults would be more plastic in their habitat preferences, as different stages of the life cycle show adaptations to different types of habitats. Moreover, whereas a fully terrestrial population may become extinct if the terrestrial conditions become unsuitable, and vice versa for an obligate neotenic species, a facultatively neotenic species may be more robust to variations in quality of terrestrial or aquatic habitats. We therefore test whether neotenic species can occupy a greater diversity of habitat types, with a specific prediction that facultatively neotenic species will occur in more types of habitat than either obligate strategy.

Here, we take a phylogenetic comparative approach to test these hypothesised drivers of the evolution of neoteny, and the consequences of its evolution for niche (habitat) breadth, in the genus *Ambystoma*. Specifically, we use phylogenetic regression techniques to explicitly test each hypothesis. We also estimate ancestral states and the rates of evolutionary transitions between metamorphosing, facultative and obligate neotenic life histories to understand the evolutionary pathways between these developmental strategies over the history of the lineage.

## 2 METHODS

### 2.1 Data collection

Maximum elevation data were taken from the IUCN Red List database (IUCN, 2022) for all species except the following; *A. barbouri* (Micheletti & Storfer, 2015), *A. bishopi*, *A. mabeei*, *A. maculatum*, *A. talpoideum*, *A. texanum*, *A. talpoideum*, *A. texanum* (observations from AmphibiaWeb 2021, checked for elevation on www.freemaptools.com/elevation‐finder.htm), *A. californiense* (Lannoo, 2005), *A. cingulatum*, (animaldiversity.org, 2022), *A. jeffersonianum* (Thompson & Gates, 1982) and *A. opacum* (Klemens, 1993). Latitudinal data (northern and southern distributional limits) were collected using the species distributions from the IUCN Red List database (IUCN, 2022). The number of habitats occupied by each species was estimated by counting the second‐level habitat types (for example 1.4 Forest ‐Temperate) listed in the IUCN Red List (IUCN, 2022) according to their habitat classification scheme. Inclusion in the *Ambystoma jeffersonianum* polyploid complex was based on information from Robertson et al. (2006), Lowcock et al. (1991) and Bogart and Lichts (1986). The presence of the species in a cave environment was based on information from Briggler (2007), Jones and Thompson (1983), Sunny et al. (2014), Gorički et al. (2019) and Ryk (2019). We did not include reports of the species from undercut riverbanks as cave‐living, as although semi‐enclosed, these represent a microhabitat within a river quite distinct from caves as relevant to our hypothesis.

Data on neoteny were collected from amphibiaweb.org and Everson et al. (2021) and coded as a categorical variable with three levels: no neoteny (metamorphosing into terrestrial adults), facultative neoteny and obligate neoteny. This initial three state variable is herein referred to as ‘frequency of neoteny’. For our analyses, we considered three further alternative variables by recoding our initial three‐state variable. The recoded variables considered were ‘neoteny’ (pooling facultative or obligate neoteny), ‘facultative neoteny’ (binary variable distinguishing facultative neoteny from both no and obligate neoteny) and obligate neoteny (binary variable distinguishing obligate neoteny from both no and facultative neoteny). We chose to use these alternative variables because it is possible that some of the proposed hypotheses might apply to, for example, obligate neoteny only, facultative neoteny or any degree of neoteny (either as if they were the same thing or in an ordered way with obligate neoteny more extreme than facultative). Hence, using our multiple coding scheme strategy enables more in‐depth evaluation of the hypotheses and under what conditions they might apply.

We downloaded 1000 phylogenetic trees for *Ambystoma* from vertlife.org (Jetz & Pyron, 2018) and calculated the maximum clade credibility tree using the mcc function of the phangorn package v2.11.1 (Schliep, 2011) in RStudio v1.3.1093 (RStudio Team, 2020). This tree was used for all comparative analyses in our study.

### 2.2 Evaluation of suitability of traits for model‐based comparative analyses

We evaluated the suitability of every categorical variable for comparative modelling by calculating the phylogenetic imbalance ratio (PIR; Gardner & Organ, 2021) using the windex package 2.02 (Arbuckle & Minter, 2015). We also calculated PIR for combinations of categorical traits that were both present (as response and explanatory variables) in a given phylogenetic generalised linear model. PIR values range between 0 and 1 and lower values indicate greater suitability of the data for modelling; although no strict cut‐offs are sensibly applicable, Gardner and Organ (2021) suggested that PIR < 0.1 is indicative of the trait(s) in question being appropriate for modelling as they are likely to be able to produce reasonable parameter estimates. We found that obligate neoteny and models including hybridisation‐induced polyploidy were unlikely to be suitable for model‐based inference (Table 1), and so we do not consider these traits further in our analyses.

**TABLE 1:** PIR values of each neotenic trait and combination of each one with categorical explanatory variables in our phylogenetic generalised linear models.

Trait(s)	PIR
Neoteny	0.03
Neoteny and Polyploidy	0.18
Neoteny and Presence in Caves	0.08
Facultative Neoteny	0.00
Facultative Neoteny and Polyploidy	0.11
Facultative Neoteny and Presence in Caves	0.06
Frequency of Neoteny	0.07
Frequency of Neoteny and Polyploidy	0.14
Frequency of Neoteny and Presence in Caves	0.10
Obligate Neoteny	0.25
Obligate Neoteny and Polyploidy	0.22
Obligate Neoteny and Presence in Caves	0.12

### 2.3 Phylogenetic generalised linear models

We fit a series of phylogenetic generalised linear models to test our hypotheses using the phylolm v2.6.2 package (Ho et al., 2016). The models test the following aforementioned ecological and geographical factors; maximum elevation, northern range limit, southern range limit, polyploidy and presence in caves. Each factor was tested as an explanatory variable in two models each, with the two neotenic variables (neoteny and facultative neoteny) suitable for modelling included as response variables. We used phylogenetic logistic regressions based on penalised likelihood estimation to predict the presence of neoteny or facultative neoteny (coded as 0 or 1 for no neoteny, and facultative/ obligate neoteny respectively). We then tested whether neoteny influences the habitat diversity available to a species by fitting a phylogenetic Poisson regression with number of habitats occupied as the response variable and our three‐state frequency of neoteny trait as the explanatory variable. Note that we run all models with a single explanatory variable per model to avoid overparameterisation, since the total diversity of the clade (32 species) would likely prevent effective parameter estimation if we attempted to fit multiple explanatory variables within a single model.

### 2.4 Ancestral state estimation

We estimated ancestral states in phytools v1.9 (Revell, 2012) for our three‐state frequency of neotenic trait and other traits which showed evidence of being related to neoteny in our phylogenetic generalised linear models. Estimation for continuous traits was carried out by maximum likelihood using the contMap function. For categorical traits, we used Bayesian stochastic mapping under an ‘all rates different’ model, based on 1000 simulations, with the root node being sampled from the conditional scaled likelihood distribution at the root. We extracted the transition rates of the estimated model for frequency of neoteny to better understand the evolutionary pathways between no, facultative and obligate neoteny.

## 3 RESULTS

**FIGURE 2:**
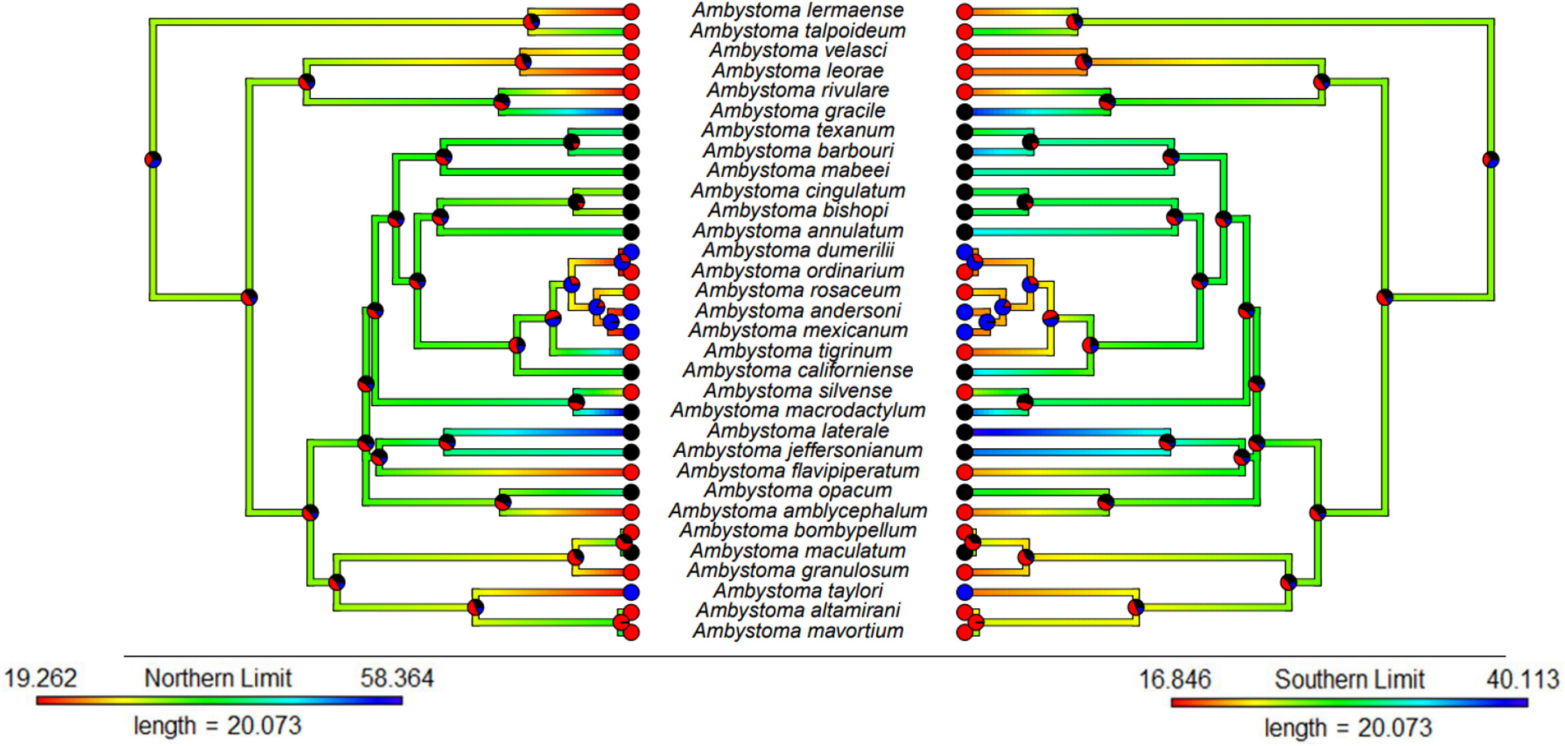
Ancestral state estimation of northern and southern range limits (shown by branch colour) and frequency of neoteny (shown as pie charts at nodes and tips representing probability of being in each state). Pie chart colours represent the following: black = no neoteny, red = facultative neoteny, blue = obligate neoteny.

**FIGURE 3:**
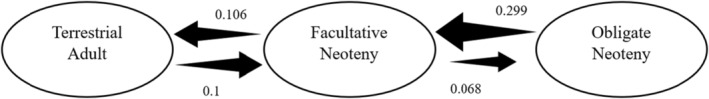
Estimated evolutionary transition rates between neotenic states of *Ambystoma* species. Despite transitions being allowed between all states, the estimated parameters strongly suggest that direct transitions between no and obligate neoteny cannot occur, instead facultative neoteny acts as an ‘evolutionary stepping stone’ between these opposite extremes. Size of arrows are proportional to the transition rate between the states they link between, and the rates themselves are given in the adjacent numbers.

**FIGURE 4:**
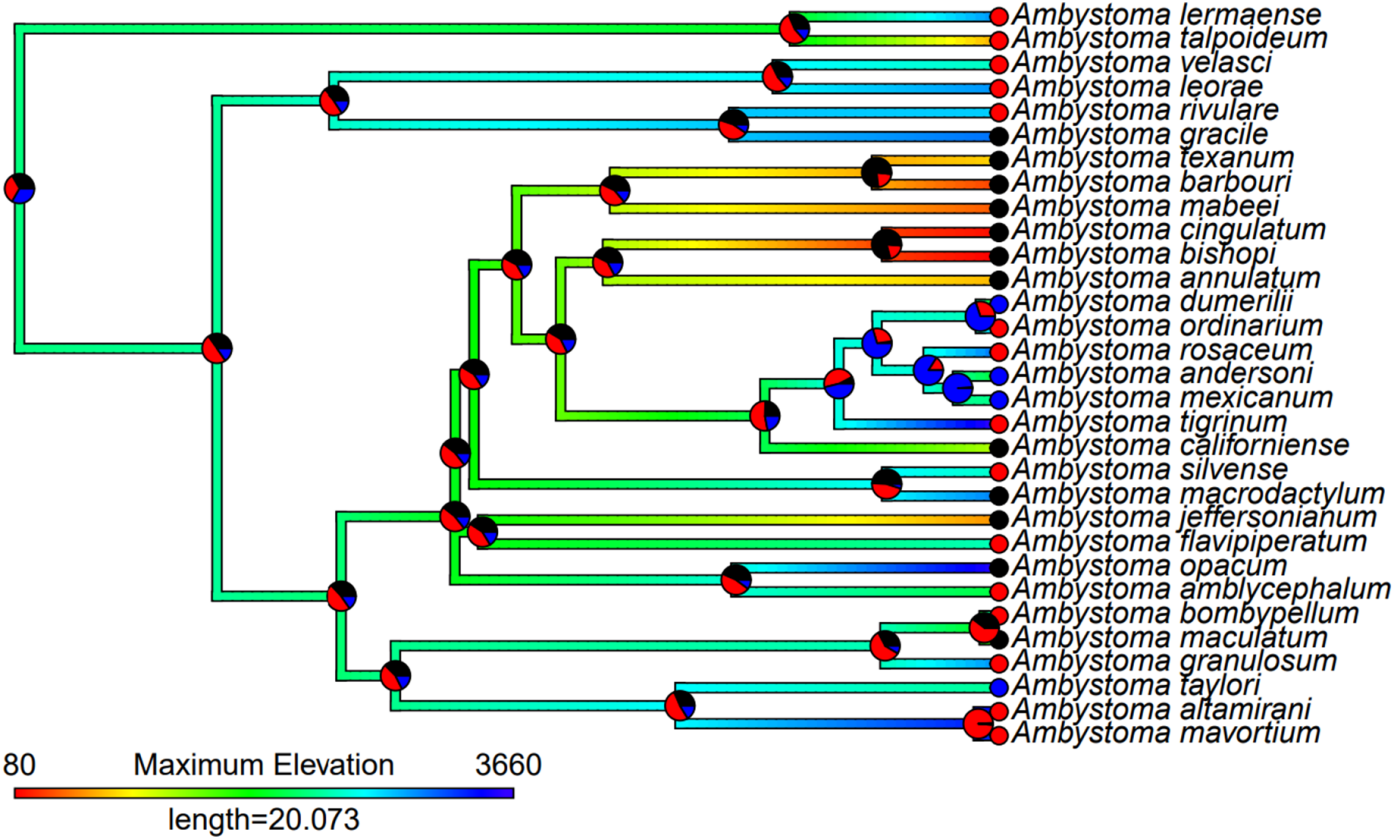
Ancestral state estimation of maximum elevation (shown by branch colour) and frequency of neoteny (shown as pie charts at nodes and tips representing probability of being in each state). Pie chart colours represent the following: black = no neoteny, red = facultative neoteny, blue = obligate neoteny. Note that *A. laterale* is absent from this phylogeny as no suitable data were available for its maximum elevational range.

Lower latitudinal distribution is associated with neoteny, such that neotenic species have both the northern and southern limits of their distributions shifted southwards relative to non‐neotenic species (Table 2; Figures 2 and 3). Considering facultative neoteny specifically, we find the same result for the southern but not the northern distributional limit, and evidence for both is weaker than for neoteny in general (Table 2). However, since this comparison combines metamorphic and obligate neotenic species to investigate any specific correlates of facultative (vs. any degree of) neoteny, this more equivocal support for latitudinal pattern is evidently driven by a more linear pattern with increasing degree of neoteny, and facultative neoteny is an intermediate state in this sequence (cf. Figure 3). Maximum elevation of neotenic species also tends to be higher than other species, although this effect is not as strong as latitude (Table 2; Figures 3 and 4). Presence in caves was not associated with neoteny in any of our models (Table 2).

**TABLE 2:** Phylogenetic generalised linear models predicting each of the neotenic variables from ecological traits.

Neotenic trait	Explanatory variable	Coefficient	SE	*z*	*p*
Neoteny	**Max. Elevation**	**0.001**	**4.763 × 10** ^**−4**^	**2.946**	**.003**
	**Northern Range limit**	**−0.078**	**0.033**	**−2.328**	**.020**
	**Southern Range limit**	**−0.291**	**0.088**	**−3.289**	**.001**
	Presence in caves	0.006	0.759	0.008	.994
Facultative Neoteny	**Max. Elevation**	**0.001**	**4.345 × 10** ^**−4**^	**2.599**	**.009**
	Northern Range limit	−0.020	0.021	−0.945	.345
	**Southern Range limit**	**−0.101**	**0.051**	**−1.974**	**.048**
	Presence in caves	0.800	0.817	0.979	.328

*Note*: Variables with significant effects at *p* ≤ .05 are in bold font.

Abbreviation: SE, standard error of coefficient.

We found strong evidence that neoteny was associated with a lower diversity of habitats (Table 3). Not only were neotenic species associated with fewer habitats than non‐neotenic species but also obligate neoteny was associated with fewer habitats than facultative neoteny.

**TABLE 3:** Phylogenetic Poisson regression predicting whether frequency of neoteny influenced the diversity of habitats a species can occupy.

Frequency of neoteny	Coefficient	SE	*z*	*p*
Intercept (Neoteny)	1.689	0.294	5.737	**9.624 × 10** ^**−9**^
Facultative Neoteny	−0.231	0.092	−2.504	**.012**
Obligate Neoteny	−0.623	0.185	−3.371	**.001**

*Note*: The intercept represents non‐neotenic species. Significant effects on habitat diversity at *p* ≤ .05 are in bold font.

Abbreviation: SE, standard error of the coefficient.

Ancestral state estimations of neoteny, latitudinal and elevational distributions contain substantial uncertainty at older nodes, but where stronger support for particular ancestral states exists these show concordant links between ecology and neoteny as indicated by our other analyses. Specifically, clades with strongly supported facultatively or obligate neotenic ancestors tend to also have more southerly distribution estimated (Figure 2), and clades with strongly supported facultatively neotenic ancestors tend to have higher maximum elevations (Figure 4).

**FIGURE 5:**
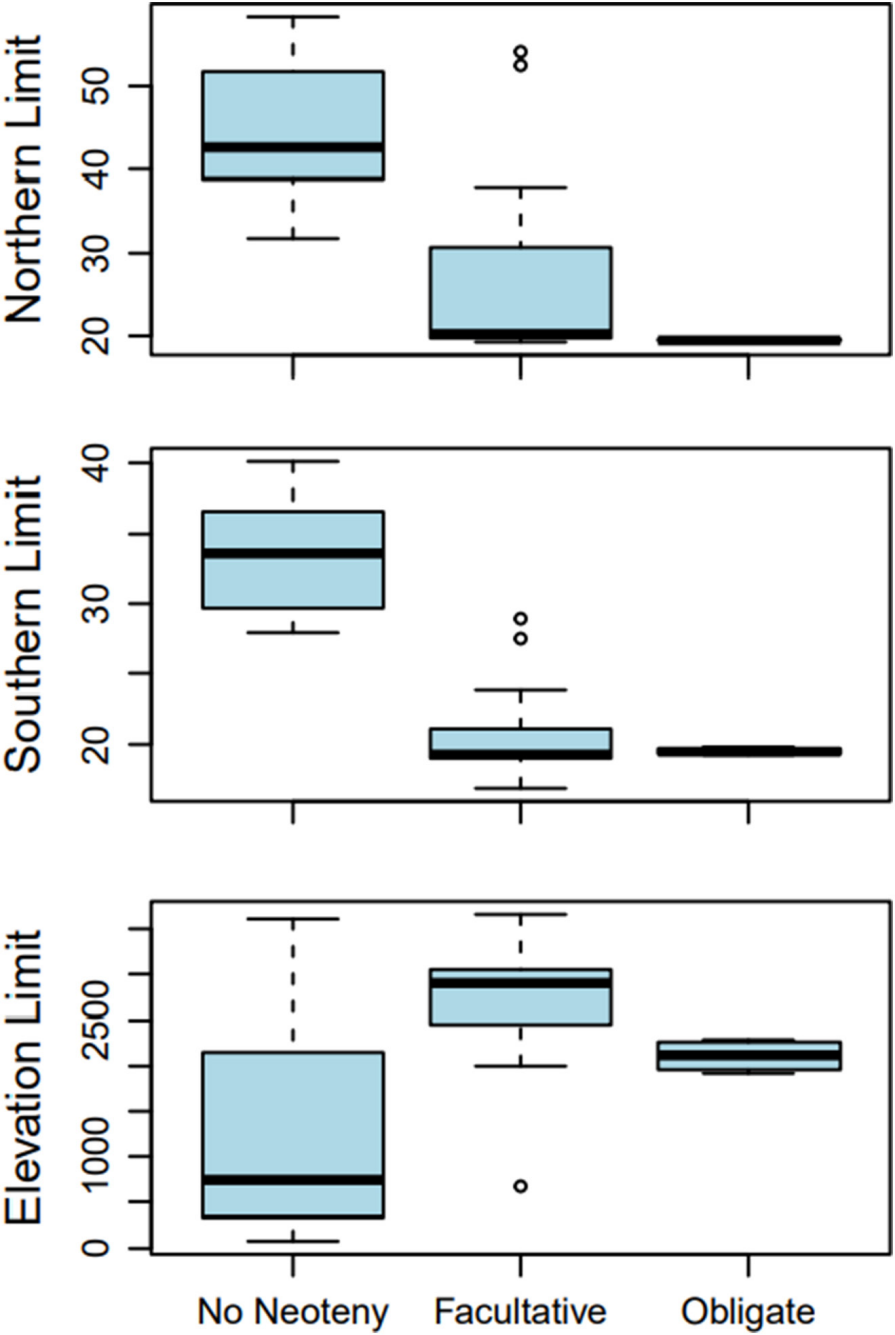
Distribution of (northern and southern) range limits and maximum elevation by neotenic state. Facultative and obligate neotenic species have similar median latitudinal distribution, but obligate species have a narrower range. In contrast, facultatively neotenic species occur at higher elevations than other species, followed by obligately neotenic species and non‐neotenic species.

The estimated transition rates for neoteny recovered facultative neoteny as an evolutionary ‘stepping stone’ between a non‐neotenic state and obligate neoteny (Figure 5). Specifically, despite the model being free to estimate direct transitions between no and obligate neoteny, the inferred transition rate was 0 in either direction between these states. While transitions between no neoteny and facultative neoteny were relatively frequent and approximately equally likely regardless of direction, transitions from facultative to obligate neoteny were less common (Figure 5). Moreover, once gained, obligate neoteny is prone to be lost (rate of loss of obligate neoteny is >4 times higher than the rate of gain), which is consistent with its rarity across the clade.

## 4 DISCUSSION

We investigated several proposed factors favouring the evolution of neoteny in salamanders, and found that it was strongly associated with lower latitudes, presumably due to reduced seasonality and possibly increased stability of aquatic habitats. We also found evidence that neotenic salamanders were associated with higher elevations. However, any effect of elevation is presumably due to isolation and/or high precipitation (similar to latitude, perhaps associated with more stable aquatic environments) rather than cooler temperatures, as temperature‐based predictions are not supported by the effect of latitude. We found no evidence that cave‐dwelling is related to neoteny (in contrast to plethodontid salamanders; Bonett et al., 2014), but were unable to test the hypothesis that hybridisation‐induced polyploidy was a factor as the phylogenetic distribution of these traits makes them unsuitable for analyses. Facultative neoteny appears to act as an evolutionary stepping stone between non‐neotenic and obligate neotenic species, with transitions directly between the latter two states unlikely to occur. Finally, we found strong evidence that neoteny limits the diversity of habitats that can be occupied, presumably imposing an ecological cost.

Some degree of neoteny is widespread across *Ambystoma* species. It occurs in 19 of the 32 extant species. Moreover, neoteny often varies between closely related taxa, despite certain clades having general tendencies towards being neotenic or not. For example, *A. ordinarium* and *A. dumerilii* diverged within the last 1 mya (Jetz & Pyron, 2018), but have evolved distinct developmental strategies; facultative and obligate neotenic respectively. This suggests that neoteny can show relatively fast evolutionary change in mole salamanders, presumably driven by strong adaption to changing abiotic conditions (Hairston et al., 2005). In keeping with this, our results find latitude as the strongest predictor of neoteny, with a narrow latitudinal range appearing to be favourable to its evolution. Although many environmental attributes vary with latitude, greater environmental stability is likely the main explanation here, resulting in less pressure for energetically expensive morphological change across the seasons (Bonett et al., 2022; Teeri & Stowe, 1976). Our results are contrary to another prediction that neoteny should be more common at the cooler temperatures of higher latitudes, due to inhibition of key hormones for metamorphosis (Moriya, 1983). Consequently, our results suggest that cool temperatures are unlikely to be the main driver, and if this also applies to the effect of elevation we recover here, the latter may also be best explained by the stability of the environment, in this case consistently high precipitation (Everson et al., 2021).

We find that metamorphic species generally occur above 30° N, with facultative species found largely from 20–30° N and obligate neotenic species being limited to a very narrow latitudinal range at the southern end of the distribution of the genus, around 20° N. This suggests that 20–30° N, and especially the most southerly end of this, provides conditions that are ‘just right’ for the evolution of neoteny, and we consequently dub this latitudinal band the ‘Neoteny Goldilocks Zone’.

One explanation for this is the reduced seasonality within the Neoteny Goldilocks Zone compared to more northerly parts of the distribution of *Ambystoma*, in that there is more stable temperature and higher rainfall (Teeri & Stowe, 1976). Such conditions may promote the evolution of neoteny by maintaining high resource abundance throughout the year and presenting a lower risk of water bodies drying up; vital for fully aquatic life cycles. Future studies using ecological niche modelling or explicit finer‐scale measures of seasonality and precipitation will help to test our idea that latitudinal patterns are driven by these environmental variables.

In most metamorphic amphibians, including many mole salamanders, development is dependent on the changing seasons, triggered by changing temperature, day length or rainfall (Bonett et al., 2022; Low, 2021; Sinai et al., 2022). The reduced seasonality of the Neoteny Goldilocks Zone compared to more northern latitudes may provide weaker cues for the timing of metamorphosis. Hence, if ecological conditions favour neoteny, it might be easier to shift away from metamorphosis if cues for the start of the process are less strong.

Metamorphosis enables the occupation of two distinct habitats (terrestrial adults vs. aquatic larvae), but comes with a substantial energetic burden to fuel the developmental changes. In environments where resources are sufficiently stable year‐round, salamanders may be able to remain in aquatic habitat, avoid paying the costs, and hence reproduce earlier and with greater energy reserves (Bonett et al., 2014). Such conditions characterise lower elevations, but harsher terrestrial environments may also result in similar selection (shifting the balance of resources away from terrestrial habitats), which may also explain the possible association with high mountainous habitat (Bonett et al., 2014). This might also suggest that, rather than being a constraint imposed by metamorphosis, our finding that neoteny is associated with fewer occupied habitat types could reflect neoteny as an adaptative specialisation to stable aquatic habitats. Hence, similar to the situation for arboreal salamanders (Baken et al., 2021), the limited latitudinal range of neotenic salamanders may be explained by the limited availability of their specialist habitat requirements.

Anthropogenic climate change has led many northern hemisphere animals shifting their distributions northwards as they track shifts in low temperature limits or escape shifting high temperature limits to the south (Milán‐García et al., 2021). The effects we found of latitude and elevation are in opposing directions in relation to temperature (higher elevations are cooler, lower latitudes are warmer) which may suggest that temperature is not tightly linked to neoteny. However, the explanation of the strong latitudinal restrictions of neotenic *Ambystoma* remains unclear, and even if temperature is not important in the evolution of neoteny, this does not imply that thermal limits on distribution are absent. Moreover, due to the narrow latitudinal range of obligate neotenic species within the Neoteny Goldilocks Zone and the requirement for stable aquatic habitats, they are typically found in relatively isolated freshwater systems. This means that shifting distributions to avoid shifting climate zones or other threats may be challenging without the ability to traverse terrestrial habitats.

Although we estimated relatively fast rates of loss of obligate neoteny in favour of facultative neoteny, these rates are on macroevolutionary timescales and may be insufficient to alleviate climate change‐induced stresses. Indeed, of the 30 *Ambystoma* species evaluated by the IUCN Red List (IUCN, 2022), 15 (50%) are threatened (six Critically Endangered, eight Endangered and one Vulnerable). Of these, 12 (80%) are at least facultatively neotenic including all six Critically Endangered species, which is proportionally higher than the 59% of at least facultatively neotenic species in our dataset. Even more starkly, the six Critically Endangered species include all four obligate neotenic species in our data set, strongly suggesting that neotenic species are in need of disproportionate conservation concern, perhaps an indirect consequence of the restricted latitudinal range of this developmental strategy.

Finally, we find that the evolution of neoteny follows a fairly constrained evolutionary pathway. The evolution of facultative neoteny from non‐neotenic populations appears to be a relatively frequent occurrence, but it is lost at a similar rate as it is gained. This suggests that mole salamanders are capable of quickly and easily evolving facultative neoteny if selection favours it. However, obligate neoteny evolves relatively rarely, and only from a facultatively neotenic intermediate, and it usually quickly lost again in favour of facultative neoteny. It is likely that the ecological flexibility of a facultative strategy is usually beneficial compared to a more specialised obligate neotenic strategy, and this may explain the relative paucity of extant species with obligate neoteny (Denoël et al., 2005).

Here, we have used data on mole salamanders (*Ambystoma*) to test previously hypothesised factors that may drive the evolution of neoteny. Unlike in some other salamander groups, we find no evidence of cave‐dwelling being related to neoteny, but we find an association of neotenic salamanders with higher elevations. Most importantly, we find strong support for a latitudinal hotspot of neotenic salamanders between 20–30° N, which we dub the Neoteny Goldilocks Zone, and the presence of obligate neotenic salamanders occurs in a particularly narrow band within this at around 20° N. We suggest that a combination of more stable aquatic habitats in this region, weaker seasonal cues to reproduction, and relatively stable food resources in this area are conducive to evolving neoteny as a specialist strategy to take advantage of reduced need for the energetically costly process of metamorphosis.

## AUTHOR CONTRIBUTIONS

**Thom A. Lyons:** Conceptualization (supporting); data curation (lead); formal analysis (lead); methodology (supporting); writing – original draft (lead); writing – review and editing (supporting). **Kevin Arbuckle:** Conceptualization (lead); formal analysis (supporting); methodology (equal); supervision (lead); validation (lead); writing – review and editing (lead).

## ACKNOWLEDGEMENTS

None.

## CONFLICT OF INTEREST STATEMENT

No competing interests.

## Data Availability

Data used in this paper are uploaded alongside this file.
