# Correction to “The neoteny goldilocks zone: The evolution of neoteny in *Ambystoma*”

**DOI:** 10.1002/ece3.70084

**Published:** 2024-08-07

Lyons, T. A., & Arbuckle, K. (2024). The neoteny goldilocks zone: The evolution of neoteny in *Ambystoma*. *Ecology and Evolution*, 14, e11240. https://doi.org/10.1002/ece3.11240

Following publication of Lyons and Arbuckle (2024), an error in the coding of a key variable (neoteny) for a single species was made known to us. Specifically, the species *Ambystoma gracile* is facultatively neotenic but was incorrectly coded as non‐neotenic in our dataset. We have corrected this error and re‐run all analyses exactly as described in the original paper to check that our results are robust to this error. In all cases the change makes only a minor quantitative, not qualitative, difference to our results and does not change the interpretations or conclusion presented in the paper. In fact, in most cases the corrected dataset strengthens our conclusions, including providing better resolution for many internal nodes in our ancestral state estimation of neoteny. Nevertheless, we here provide the corrected figures and tables to ensure these are available.

The one slight change necessary to our descriptions of results relates to the transition rate estimates (shown in Figure 3 in the original manuscript and below). Our key results from this analysis remain correct as originally described, but one sentence describing the relation between some transition rates in the Results needs updating. Originally we wrote the following in the Results concerning the transition rate estimates (sentence requiring correction in italics):

“The estimated transition rates for neoteny recovered facultative neoteny as an evolutionary ‘stepping stone’ between a non‐neotenic state and obligate neoteny (Figure 5). Specifically, despite the model being free to estimate direct transitions between no and obligate neoteny, the inferred transition rate was 0 in either direction between these states. *While transitions between no neoteny and facultative neoteny were relatively frequent and approximately equally likely regardless of direction, transitions from facultative to obligate neoteny were less common*. Moreover, once gained, obligate neoteny is prone to be lost (rate of loss of obligate neoteny is >4 times higher than the rate of gain), which is consistent with its rarity across the clade.”

The italicised sentence should now read as follows:

“Transitions between no neoteny and facultative neoteny were approximately equally likely (slightly more likely from no to facultative neoteny), whereas transitions from facultative to obligate neoteny were slightly more common, suggesting a tendency towards increasing degree of neoteny over time in lineages.”

Note that this does not alter our discussion as our key results were the non‐italicised sections, that is, that neoteny evolution is constrained to follow a ‘stepping stone’ model and obligate neoteny is lost at a rate > four times higher than it is gained. Hence despite the corrections given here, the interpretation, discussion and conclusions in the original manuscript remain unchanged.1, 2, 3, 2, 3, 4, 5

**TABLE 1:** PIR values of each neotenic trait and combination of each one with categorical explanatory variables in our phylogenetic generalised linear models. Values in square brackets are values in the original paper (before correction of datapoint for *Ambystoma gracile*).

Trait(s)	PIR
Neoteny	0.04 [0.03]
Neoteny and polyploidy	0.21 [0.18]
Neoteny and presence in caves	0.09 [0.08]
Facultative neoteny	0.01 [0.00]
Facultative neoteny and polyploidy	0.13 [0.11]
Facultative neoteny and presence in caves	0.06 [0.06]
Frequency of neoteny	0.08 [0.07]
Frequency of neoteny and polyploidy	0.16 [0.14]
Frequency of neoteny and presence in caves	0.12 [0.10]
Obligate neoteny	0.25 [0.25]
Obligate neoteny and polyploidy	0.20 [0.22]
Obligate neoteny and presence in caves	0.20 [0.12]

**TABLE 2:** Phylogenetic generalised linear models predicting each of the neotenic variables from ecological traits. SE, standard error of coefficient. Variables with significant effects at *p* ≤ .05 are in bold font. Values in square brackets are values in the original paper (before correction of datapoint for *Ambystoma gracile*).

Neotenic trait	Explanatory variable	Coefficient	SE	*z*	*p*
Neoteny	**Max. elevation**	**0.002 [0.001]**	**5.187 × 10** ^**−4**^ **[4.763 × 10** ^**−4**^ **]**	**2.998 [2.946]**	**.003 [.003]**
	**Northern range limit**	**−0.086 [−0.078]**	**0.037 [0.033]**	**−2.287 [−2.328]**	**.022 [.020]**
	**Southern range limit**	**−0.337 [−0.291]**	**0.128 [0.088]**	**−2.628 [−3.289]**	**.009 [.001]**
	Presence in caves	−0.336 [0.006]	0.692 [0.759]	−0.485 [0.008]	.628 [.994]
Facultative neoteny	**Max. elevation**	**0.002 [0.001]**	**5.021 × 10** ^**−4**^ **[4.345 × 10** ^**−4**^ **]**	**2.991 [2.599]**	**.003 [.009]**
	Northern range limit	−0.045 [−0.020]	0.025 [0.021]	−1.815 [−0.945]	.070 [.345]
	**Southern range limit**	**−0.179 [−0.101]**	**0.067 [0.051]**	**−2.658 [−1.974]**	**.008 [.048]**
	Presence in caves	0.386 [0.800]	0.912 [0.817]	0.424 [0.979]	.672 [.328]

**TABLE 3:** Phylogenetic Poisson regression predicting whether frequency of neoteny influenced the diversity of habitats a species can occupy. The intercept represents non‐neotenic species. SE, standard error of the coefficient. Significant effects on habitat diversity at *p* ≤ .05 are in bold font. The reference level (intercept) is no neoteny, and values in square brackets are values in the original paper (before correction of datapoint for *Ambystoma gracile*).

Frequency of neoteny	Coefficient	SE	*z*	*p*
**Intercept**	**1.696 [1.689]**	**0.291 [0.294]**	**5.830 [5.737]**	**5.557 × 10** ^**−9**^ **[9.624 × 10** ^**−9**^ **]**
**Facultative neoteny**	**−0.217 [−0.231]**	**0.091 [0.092]**	**−2.398 [−2.504]**	**.017 [.012]**
**Obligate neoteny**	**−0.606 [−0.623]**	**0.179 [0.185]**	**−3.389 [−3.371]**	**.001 [.001]**

**FIGURE 2:**
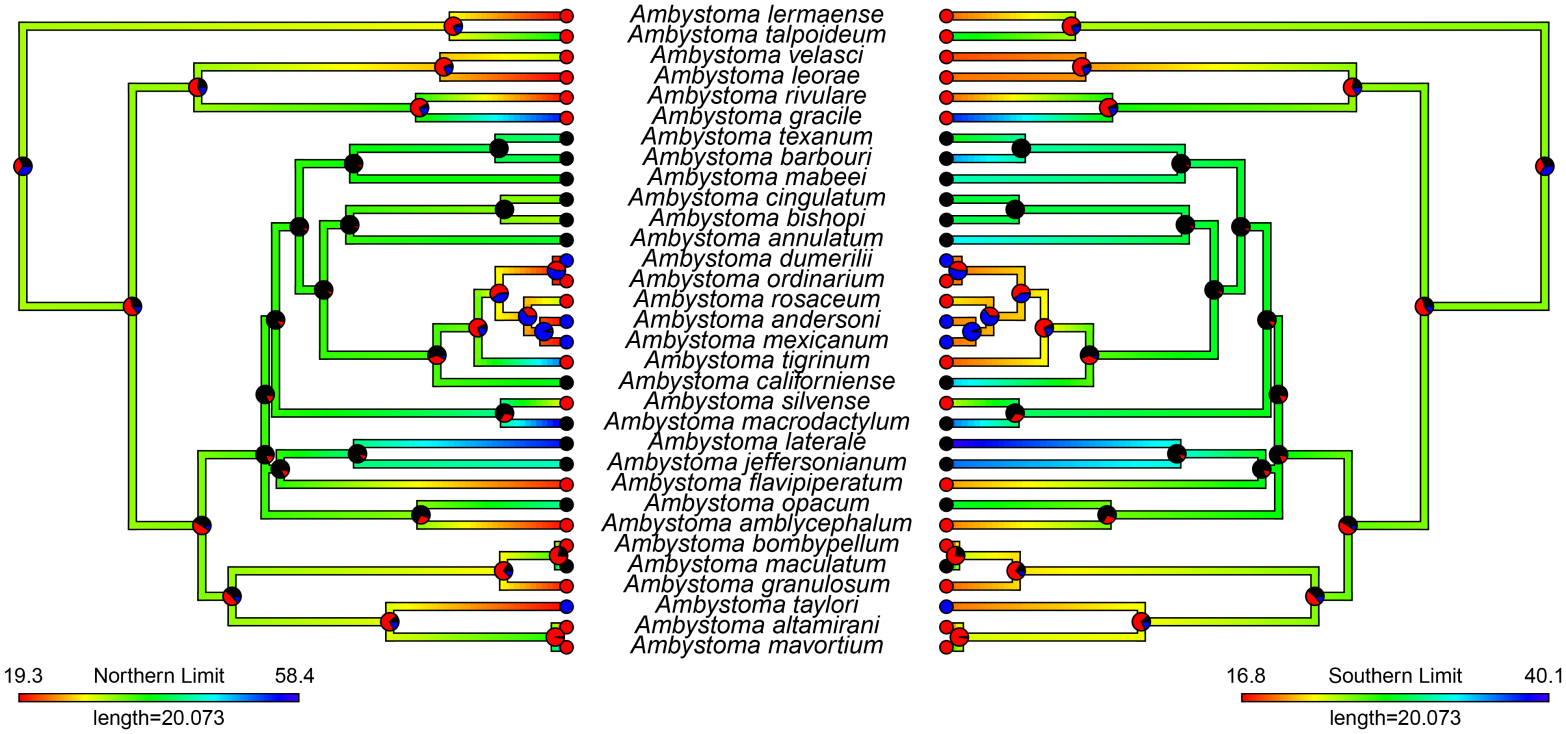
Ancestral state estimation of northern and southern range limits (shown by branch colour) and frequency of neoteny (shown as pie charts at nodes and tips representing probability of being in each state). Pie chart colours represent the following: Black = no neoteny, red = facultative neoteny, blue = obligate neoteny.

**FIGURE 3:**
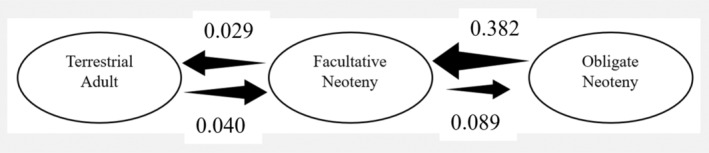
Estimated evolutionary transition rates between neotenic states of *Ambystoma* species. Despite transitions being allowed between all states, the estimated parameters strongly suggest that direct transitions between no and obligate neoteny cannot occur, instead facultative neoteny acts as an ‘evolutionary stepping stone’ between these opposite extremes. Transition rates are given next to their relevant arrows indicating transitions.

**FIGURE 4:**
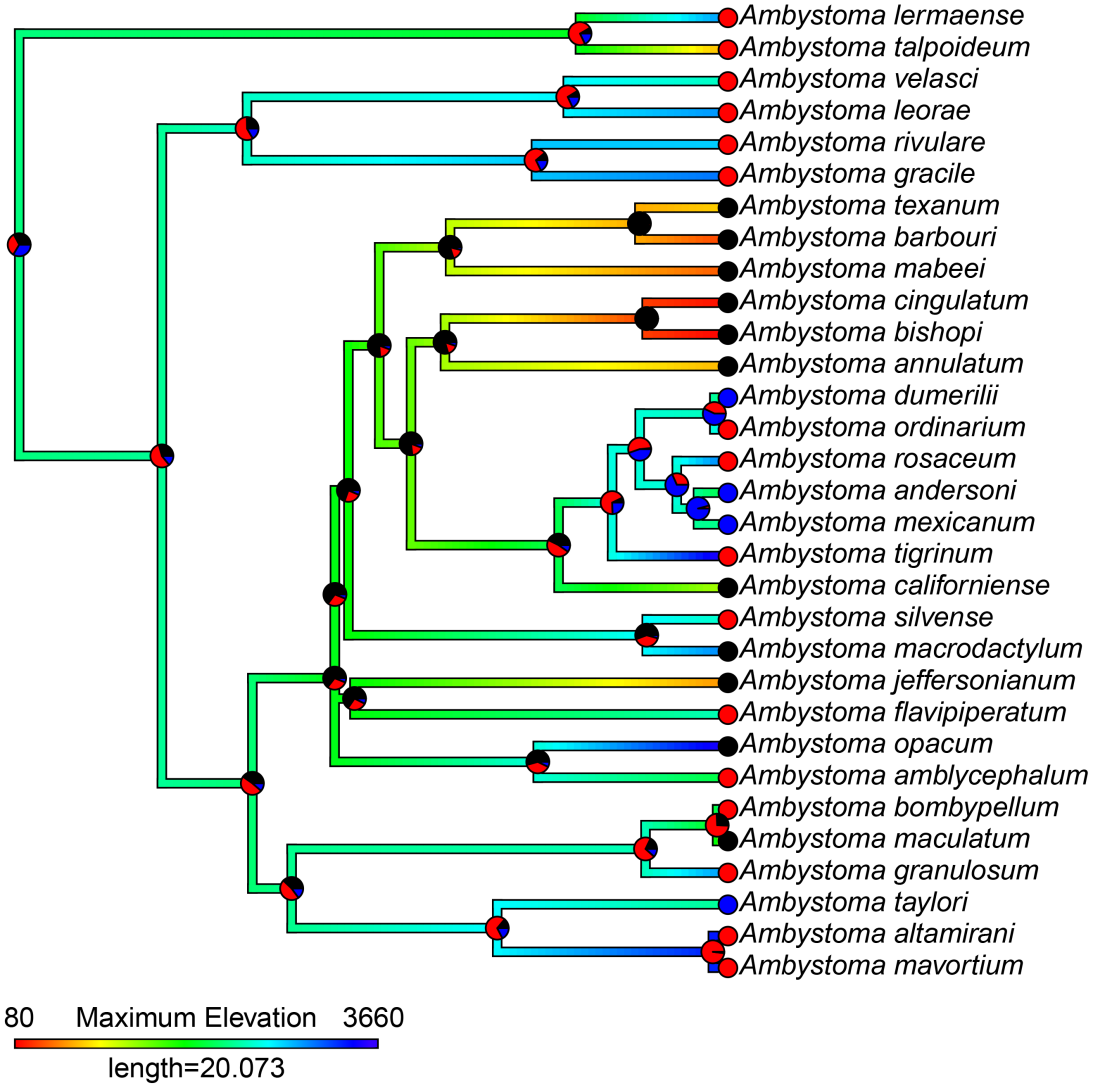
Ancestral state estimation of maximum elevation (shown by branch colour) and frequency of neoteny (shown as pie charts at nodes and tips representing probability of being in each state). Pie chart colours represent the following: Black = no neoteny, red = facultative neoteny, blue = obligate neoteny. Note that *A. laterale* is absent from this phylogeny as no suitable data was available for its maximum elevational range.

**FIGURE 5:**
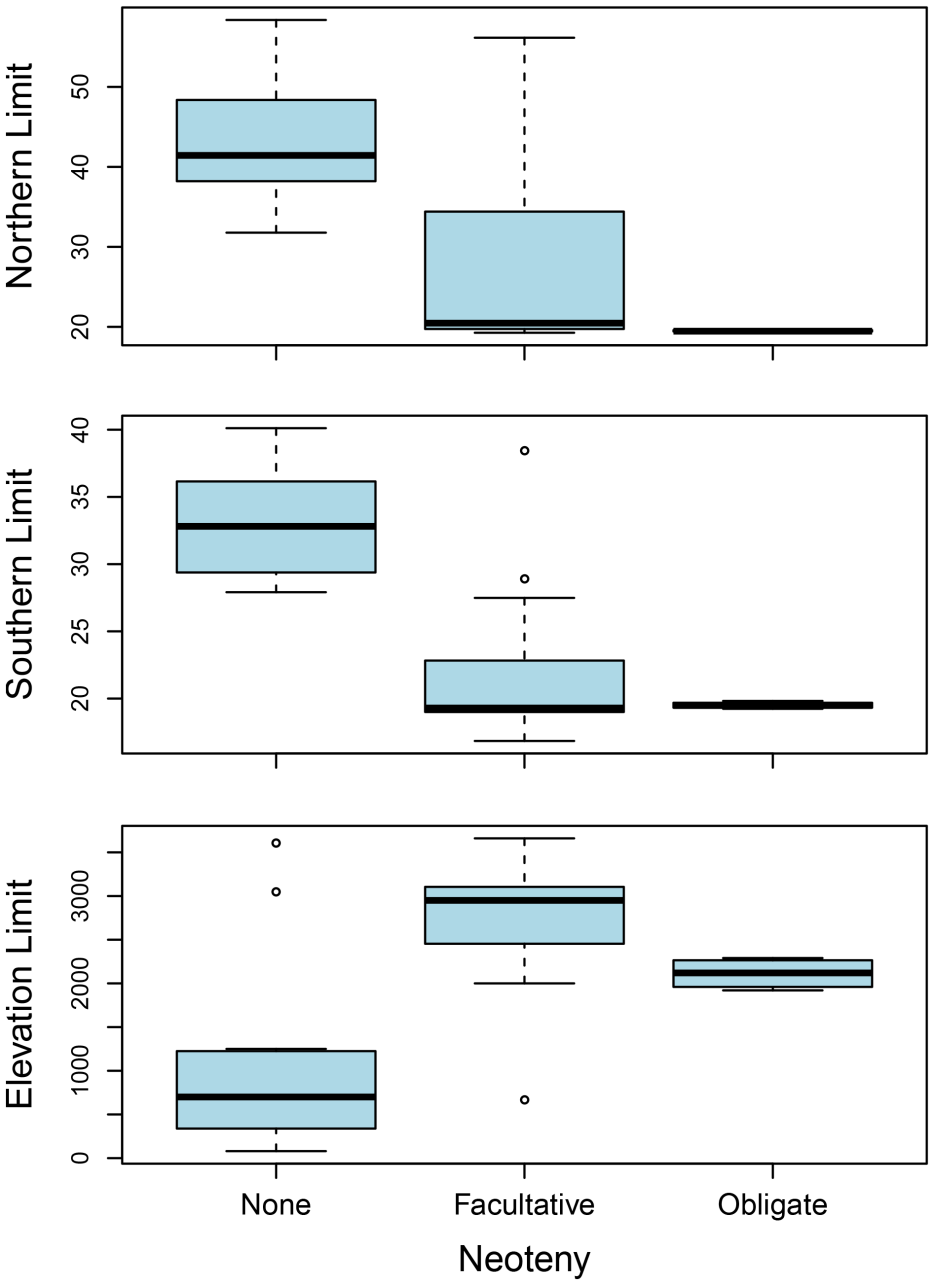
Distribution of (northern and southern) range limits and maximum elevation by neotenic state. Facultative and obligate neotenic species have similar median latitudinal distribution, but obligate species have a narrower range. In contrast, facultatively neotenic species occur at higher elevations than other species, followed by obligately neotenic species and non‐neotenic species.

